# Antinuclear antibodies positive acute nonfulminant hepatitis A associated with acute renal failure and hives: a case report

**DOI:** 10.1097/MS9.0000000000000317

**Published:** 2023-03-27

**Authors:** Marwa Kliea, Mohammad Alsultan, Kassem Basha

**Affiliations:** Departments of a Neurology; b Nephrology, Faculty of Medicine, Al Assad and Al Mouwasat University Hospitals; c Department of Nephrology , Faculty of Medicine, Al Mouwasat University Hospitals, Damascus University, Damascus, Syria

**Keywords:** acute hepatitis A, hepatitis A virus, acute renal failure, acute kidney injury, Hives, antinuclear antibodies

## Abstract

Acute hepatitis A (AHA) is a self-limited illness. While the prognosis for hepatitis A is generally good, complications with acute renal failure can have a negative impact.

**Presentation of case:**

A 60-year-old male was admitted due to fever and malaise for a week, accompanied by jaundice and reduced urine output over the past 3 days. The patient was exhausted with icteric skin and sclera, dark urine, bilateral pretibial grade II pitting edema, and a urinary output of nearly 1 l/day. Laboratory findings on admission showed acute liver injury and acute kidney injury with positive hepatitis A virus immunoglobulin M. Liver and kidney function tests were augmented gradually aligned with oliguria. Thereafter, the patient had an itchy rash on his back and abdomen. Screening for immune diseases came back negative except for positive antinuclear antibodies. The authors continued conservative management with dialysis, diuretics, and restricted hydration. After five hemodialysis sessions, urinary output improved and liver function tests were improved, however, kidney function tests were slowly improved. One month later, serum creatinine was reduced to 1.4 mg/dl, and 2 months later, it was 1.1 mg/dl.

**Conclusion:**

The authors experienced a rare case of nonfulminant AHA that resulted in severe acute renal failure and needed dialysis. Several hypotheses had been postulated for AHA-related nephropathy; however, hyperbilirubinemia-induced acute tubular necrosis was the most acceptable theory in the patient. Since AHA associated with antinuclear antibodies positive and hives rash could confuse the diagnosis, clinicians should consider extrahepatic manifestations associated with hepatitis A virus infection in such conditions after excluding immune disorders.

## Introduction

Highlights

- Nonfulminant acute hepatitis A could cause severe acute renal failure.
- Hyperbilirubinemia-induced acute tubular necrosis was the most acceptable theory for acute hepatitis A-related nephropathy.
- Acute hepatitis A associated with antinuclear antibodies that are positive and a hives rash could confuse the diagnosis.
- Clinicians should consider extrahepatic manifestations associated with hepatitis A virus infection in such conditions after excluding immune disorders.

Acute hepatitis A (AHA) infection is caused by the hepatitis A virus (HAV). Humans are the only known reservoir. HAV infection is usually a self-limited illness that does not become chronic. Fulminant hepatic failure occurs in less than 1% of cases^1^. Symptomatic illness due to HAV occurs in more than 70% of adults^2^. Several extrahepatic manifestations associated with HAV infection have been described; the most common extrahepatic manifestations include evanescent rash (hives) and arthralgia^2^. Less common manifestations related to immune complexes such as leukocytoclastic vasculitis, arthritis, glomerulonephritis, and cryoglobulinemia, have been reported^3^,^4^. Although acute kidney injury (AKI) can develop in 63.4 and 38.8% required renal replacement therapy at any time prior to acute liver failure (ALF) recovery, the development of acute renal failure (ARF) is not a common complication of nonfulminant AHA^5^,^6^. While the prognosis of HAV is generally good, complications with ARF can have a negative impact^7^.

Here we describe a patient who developed two rare complications in the context of AHA: ARF and a hives rash. Although the evanescent rash is the most common extrahepatic manifestation, renal failure is a rare manifestation, and this is the first case in the literature to combine renal failure and hives associated with HAV with a favorable outcome. This case report is in accordance with the Declaration of Helsinki and in line with the SCARE guidelines^8^.

## Case presentation

A 60-year-old male was admitted to the Emergency Department of Al-Mouwasat University Hospital due to fever, malaise for a week accompanied by jaundice, and reduced urine output over the past 3 days. His medical history included diabetes mellitus (DM) type 2 controlled by (gliclazide 60 mg/day, metformin 1000 mg/day), hypertension controlled by losartan/hydrochlorothiazide (50/12.5/once daily), ischemic heart disease, and recurrent nephrolithiasis.

On physical examination; weight: 120 kg, blood pressure=140/70 mm Hg, heart rate=90/min, respiratory rate=24/min, oxygen saturation=98% in air room. The patient was exhausted with icteric skin and sclera, dark urine, bilateral pretibial grade II pitting edema, and a urinary output of nearly 1 l/day.

Laboratory findings on admission (Table 1) showed liver injury and renal failure, while the last known creatinine (Cr) level was 1.3 mg/dl. Renal ultrasound showed normal sized kidneys; 12.3 cm for right kidney and 13.6 cm for left kidney, normal cortical thickness; 1.2 cm for right kidney and 1 cm for left kidney, and loss of corticomedullary differentiations. Renal vessels were intact and continuous to the cortex without signs for vasculitis. Inferior vena cava measured 1.5 cm and collapse in inspiration.

**Table 1:** Laboratory tests and management from admission to complete recovery

Day	Laboratories and management on admission																Manage	
D1 (out-patient tests)	WBC	RBC	HB	PLT	Cr	ALT	AST	TB									febuxostat+colchicine+HD (first)	
	8	4.5	15	219	6.3	909		13.9										
	DB	Widal	Wright	CRP														
	9.1	Neg	Neg	30*														
D2	WBC	HB	PLT	Ur	Cr	TB	DB	ALT										
	8.1	15	259	200	11.9	17	11.6	673										
	AST	UA	LDH	CK	Ca	ALB	P	PT										
	134	20.8	305	75	7.6	3	7.4	50%										
	INR	PH	Pco2	HCO3	HAV IgM‡	HBS Ag	Anti-HCV	UO†										
	1.4	7.4	30	15	8.6	Neg	Neg	700										
	SG	Protein	Hb	RBC (cell/HPF)	WBC (cell/HPF)	Microscopy	PCR											
	1.001	+++	+++	>200	2–4	80% dysmorphic	2.2											
D 3–5	IgG	IgM	IgA	C4	C3	Anti-dsDNA	ANA	UO									HD (second+third)+ diuretics	
	1286	228	299	9.3	85	Neg	1/80	100										
	Ur	Cr	Ast	Alt	TB	DB	PH	Hco3										
	196	14.5	105	511	18	12	7.30	15										
D 10	Ur	Cr	Ast	Alt	TB	DB	UO†										HD (fourth+fifth)+diuretics	
	171	13	46	103	5.8	4	700											
D14	Ur	Cr	Ast	Alt	TB	DB	UO†										Discharged+diuretics	
	150	12	49	45	2.7	1.8	2500											
Monitoring tests after discharged																		
D1			D2			D3				D5		D7		D9		D12		
Ur	Cr	UO	Ur	Cr	UO	Ur	Cr	UO	TB/DB	Ur	Cr	Ur	Cr	Ur	Cr	Ur	Cr	TB
228	9	2.5 l	210	7.6	4 l	177	6.2	4–5 l	1.8/1.5	119	3.4	59	1.8	33	1.4	33	1.3	1.2

* CRP normal range (up to 6 mg/l).

† UO in ml/day.

‡ HAV IgM normal range less than 1 (index).

ALB, albumin; ALT, alanine transaminase; ANA, antinuclear antibody; anti-HCV Ab, antihepatitis C antibody; anti-dsDNA, antidouble stranded DNA; AST, aspartate aminotransferase; C3–4, complements; Ca, calcium; CK, creatine kinase; Cr ratio in random urine; Cr, creatinine; CRP, C-reactive protein; D, day; DB, direct bilirubin; HAV IgM, hepatitis A virus (HAV)-IgM antibody; HB, hemoglobin; HBS Ag, hepatitis B antigen; HD, hemodialysis; HPF, high-power field; HT, hematocrit; INR, international normalized ratio; LDH, lactate dehydrogenase; neg, negative; P, phosphorus; PCR, protein; PLT, platelets; PT, prothrombin time; RBC, red blood cells; SG, specific gravity; TB, total bilirubin; UA, uric acid; UO, urinary output; Ur, urea; WBC, white blood count.

On day 2 of admission, liver and kidney function tests were augmented, which showed an increase in total bilirubin to 18 mg/dl, and an increase in Cr to 10 mg/dl aligned with reduced urine output (700 ml/day) (Table 1). Tests for viral hepatitis confirmed HAV infection [immunoglobulin (Ig)M=8] (Table 1). We stopped drugs used for hypertension and DM2 because the patient developed hypoglycemia and blood pressure dropped to ~100–110/60–50 mm Hg. We prescribed febuxostat with colchicine for hyperuricemia.

On the third day, the patient got worse, with oliguria (about 100 ml/day) and worsening dyspnea being noticed despite adequate hydration and diuretic use thereafter, in line with fluid administration guidelines for critical conditions^9^. Here the first emergent hemodialysis (HD) session was obtained.

**Figure 1:**
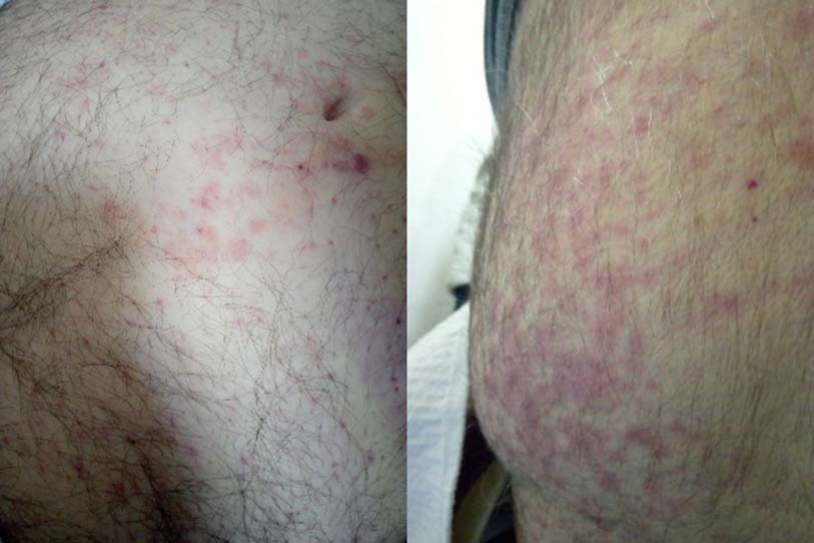
Hives rash: a maculopapular rash on an erythematous base on the abdomen (left image) and on the back (right image).

On the fifth day, the patient had an itchy rash on his back and abdomen (Fig. 1). Screening for immune diseases came back negative (Table 1). Oliguria and generalized edema obligated a second and third session of HD (Table 1).

We continued conservative management with dialysis, diuretics, and restricted hydration. After five HD sessions, his urinary output improved and reached ~1.5 l/day with diuretics and adequate hydration. Liver function tests were improved; however, kidney function tests were slowly improved (Table 1). At this point, the patient was discharged on his own responsibility, provided that he undertook daily monitoring of his vital signs and laboratory tests with a contact by daily phone calls (Table 1).

Nine days after discharge, Cr was reduced to 1.4 mg/dl, and 1 month later it was 1.1 mg/dl (Table 1).

## Discussion

Although ARF is not uncommon in patients with fulminant hepatitis, it has been recognized as a rare complication of nonfulminant AHA^5^,^6^. ARF associated with nonfulminant AHA has been infrequently reported, with only about 50 cases to our knowledge^10^. Another analysis of 208 hepatitis A patients showed ARF in three fulminant hepatitis cases and 15 nonfulminant hepatitis were also complicated with ARF^11^. Moreover, hepatitis A patients with ARF had higher alanine transaminase, peak total bilirubin, and more prolonged prothrombin time compared with patients without ARF. In this study, DM patients were more prevalent to develop ARF, which might be related to underlying renal dysfunction associated with diabetes^7^. Other hepatitis viruses like B and C are known to cause well-known nephropathies. Contrastingly, hepatitis A-related nephropathy has no definite pathogenesis, meanwhile, several hypotheses have been postulated^7^.

First, the prerenal hypothesis according to acute hepatitis with fever, anorexia, vomiting, and reduced oral intake can cause intravascular volume depletion and activation of the rennin–angiotensin–aldosterone system, both of which can compromise effective renal perfusion^12^,^13^. In our condition, the prerenal azotemia was excluded by the clinical status, where we gave fluids first, followed by diuretic use when the edema and plethoric inferior vena cava were observed.

Second, hepatitis A viral overlap the immune complex and multisystem involvement has been mentioned like kidney, joints, vessels, and skin. Immunofluorescence microscopy revealed mesangial deposits of IgG and IgM and capillary loop deposits of IgA and C3, although there was no antigen identified, in addition, some kidney biopsies have revealed various immune complex depositions, including IgG, IgA, IgM, C3, and Clq. Evanescent rash in about 14% of cases associated with hepatitis A (hives) might cause by also deposition of immune complexes, cutaneous vasculitis, or cryoglobulinemia^4^,^14^.

In this case, renal impairment is accompanied by dysmorphic red blood cells in urine microscopy, which consist of glomerular injury. In addition to dermal manifestation, this may be the proposed etiology in our patient, so we were motivated to screen for immune disorders. We started by screening tests like antinuclear antibodies (ANA) and complements, and when they were positive, we did other tests; however, they were all negative and no other diagnosis was made. Similarly, a prospective study found that ANAs were detected frequently in 42.4% of AHA patients and transiently in AHA patients, especially after the peak-alanine transaminase day, and were reversed after about 3 months^15^. The proportion of ANA-positive patients varied significantly with AHA status (4.7% during the prodromal period vs. 52.1% during the icteric or recovery period), although the presence of ANA did not have a significant effect on manifestations, complications, morbidity, and mortality. So, we did not repeat ANA, when there was no evidence in affected the outcome^15^.

Third, hyperbilirubinemia induces a kidney injury; when hepatic dysfunction occurs, the kidney becomes the main excretory organ for the retained bile substances, where the natural excretory route of bile is blocked^16^. Hyperbilirubinemia may be the major factor in the ARF associated with hepatitis A infection. These bile salts may cause direct toxic effects on cell function, such as that of the tubular epithelium, which can produce acute tubular necrosis or tubulointerstitial nephritis^17^,^18^. Also, the reduction in systemic vascular resistance or shunting of the blood away from the renal cortex predisposes to renal vasoconstriction^12^,^19^. In addition, left ventricular contractility impairment can compromise effective renal blood flow as well^12^. On the other hand, endotoxemia induced by acute hepatitis seems to be an important indirect mechanism of renal injury, including clotting system abnormalities, hemodynamic disturbances, inflammation, and oxidant injury^12^. Lipopolysaccharide (LPS), the most important mediator in the systemic response induced by endotoxemia, can induce AKI through amplifying the inflammatory response and causing direct damage to renal tissue and cells^12^. Also, LPS activates vascular endothelial cells and leukocytes, causing the secretion of a large number of inflammatory mediators that result in indirect and direct renal injury^12^. On the other hand, LPS acts directly on renal tubular epithelial cell and renal endothelial cells, resulting in renal tubular damage^12^.

In our patient, both bilirubin and Cr concentrations reached a peak within ~10 days of presentation. Dialysis can decrease bilirubin by some levels; thereafter, we noticed lowering bilirubinemia concurrently with improvement in renal failure. The AKI regression with conservative management after resolving hepatic injury, in addition to previous reports of AKI regression with dialysis and plasmapheresis^17^, makes hyperbilirubinemia-induced acute tubular necrosis the most acceptable theory.

We could not perform a kidney biopsy, because the patient’s weight was high (about 120 kg) and progressive dyspnea prevented him from lying on his abdomen. Also, we did not motivate to obtain the biopsy after status improvement because of a good prognosis based on reviewing the medical literature aligned with renal function improvement with conservation management.

## Conclusions

We experienced a rare case of nonfulminant AHA that resulted in severe ARF that needed dialysis and a hive rash. Since AHA associated with ANA positivity and rash could confuse the diagnosis, clinicians should consider extrahepatic manifestations associated with HAV infection in such conditions after excluding immune disorders.

## Ethical approval

Written informed consent was obtained from the patient for the publication of this case report and accompanying images, in line with local ethical approval requirements and in accordance with the Helsinki Declaration.

## Consent

Written informed consent was obtained from the patient for the publication of this case report and accompanying images.

## Sources of funding

None declared.

## Author contributions

M.K. writes the manuscript, literature search, article corrections, and patient follow-up. M.A. writes the manuscript, literature search, submit the article, and patient follow-up. Professor K.B. makes article corrections, supervisor, and follow-up of the patient.

## Conflicts of interest disclosure

None declared.

## Research registration unique identifying number (UIN)

1. Name of the registry: NA.
2. Unique identifying number or registration ID: NA.
3. Hyperlink to your specific registration (must be publicly accessible and will be checked): NA.

## Guarantor

The corresponding author is the guarantor of this manuscript.

## Provenance and peer review

Not commissioned, externally peer-reviewed.
